# The Coincidence Between Increasing Age, Immunosuppression, and the Incidence of Patients With Glioblastoma

**DOI:** 10.3389/fphar.2019.00200

**Published:** 2019-03-27

**Authors:** Erik Ladomersky, Denise M. Scholtens, Masha Kocherginsky, Elizabeth A. Hibler, Elizabeth T. Bartom, Sebastian Otto-Meyer, Lijie Zhai, Kristen L. Lauing, Jaehyuk Choi, Jeffrey A. Sosman, Jennifer D. Wu, Bin Zhang, Rimas V. Lukas, Derek A. Wainwright

**Affiliations:** ^1^Department of Neurological Surgery, Northwestern University Feinberg School of Medicine, Chicago, IL, United States; ^2^Department of Preventive Medicine, Northwestern University Feinberg School of Medicine, Chicago, IL, United States; ^3^Department of Obstetrics and Gynecology, Northwestern University Feinberg School of Medicine, Chicago, IL, United States; ^4^Department of Biochemistry and Molecular Genetics, Northwestern University Feinberg School of Medicine, Chicago, IL, United States; ^5^Department of Dermatology, Northwestern University Feinberg School of Medicine, Chicago, IL, United States; ^6^Department of Medicine-Hematology and Oncology, Northwestern University Feinberg School of Medicine, Chicago, IL, United States; ^7^Department of Urology, Northwestern University Feinberg School of Medicine, Chicago, IL, United States; ^8^Department of Microbiology-Immunology, Northwestern University Feinberg School of Medicine, Chicago, IL, United States; ^9^Robert H. Lurie Comprehensive Cancer Center, Northwestern University Feinberg School of Medicine, Chicago, IL, United States; ^10^Department of Neurology, Northwestern University Feinberg School of Medicine, Chicago, IL, United States

**Keywords:** aging, biomarker, IDO, immunosuppression, PD-L1, immunotherapy, Treg, IDH

## Abstract

**Background:** Glioblastoma (GBM) is the most aggressive primary brain tumor in adults and is associated with a median overall survival (mOS) of 16–21 months. Our previous work found a negative association between advanced aging and the survival benefit after treatment with immunotherapy in an experimental brain tumor model. Given the recent phase III clinical success of immunotherapy in patients with many types of cancer, but not for patients with GBM, we hypothesize that aging enhances immunosuppression in the brain and contributes to the lack of efficacy for immunotherapy to improve mOS in patients with malignant glioma. Herein, we compare epidemiological data for the incidence and mortality of patients with central nervous system (CNS) cancers, in addition to immune-related gene expression in the normal human brain, as well as peripheral blood immunological changes across the adult lifespan.

**Methods:** Data were extracted from the National Cancer Institute’s surveillance, epidemiology, and end results (SEER)-, the Broad Institute’s Genotype Tissue Expression project (GTEx)-, and the University of California San Francisco’s 10k Immunomes-databases and analyzed for associations with aging.

**Results:** The proportion of elderly individuals, defined as ≥65 years of age, has predominantly increased for more than 100 years in the United States. Over time, the rise in elderly United States citizens has correlated with an increased incidence and mortality rate associated with primary brain and other CNS cancer. With advanced aging, human mRNA expression for factors associated with immunoregulation including immunosuppressive indoleamine 2,3 dioxygenase 1 (IDO) and programmed death-ligand 1 (PD-L1), as well as the dendritic cell surface marker, CD11c, increase in the brain of normal human subjects, coincident with increased circulating immunosuppressive Tregs and decreased cytolytic CD8^+^ T cells in the peripheral blood. Strikingly, these changes are maximally pronounced in the 60–69 year old group; consistent with the median age of a diagnosis for GBM.

**Conclusion:** These data demonstrate a significant association between normal human aging and increased immunosuppression in the circulation and CNS; particularly late in life. Our data raise several hypotheses including that, aging: (i) progressively suppresses normal immunosurveillance and thereby contributes to GBM cell initiation and/or outgrowth; (ii) decreases immunotherapeutic efficacy against malignant glioma.

## Introduction

Glioblastoma (GBM) is the most common primary malignant brain tumor in adults. Despite the aggressive standard of care regimen that includes maximal surgical resection followed by radiation therapy and chemotherapy with temozolomide, and more recently tumor treating fields, the median overall survival (mOS) remains at 16–21 months post-diagnosis, with just 43% of patients surviving for 2 years post-diagnosis (Stupp et al., 2005, 2009, 2017; Johnson and O’Neill, 2012). A major factor contributing to the poor GBM patient prognosis is the potent immunosuppression, found systemically and locally in the brain tumor microenvironment (Chongsathidkiet et al., 2018). High intratumoral expression of immunosuppressive mediators including programmed cell death protein-1 (PD-1) and indoleamine 2,3 dioxygenase 1 (IDO), is prognostic for decreased GBM patient survival (Wainwright et al., 2012; Nduom et al., 2016; Zhai et al., 2017). Immune checkpoint inhibitor treatment has demonstrated a survival benefit in patients with non-small cell lung carcinoma (Antonia et al., 2016), renal cell cancer (Motzer et al., 2015), end-stage melanoma (Larkin et al., 2015), and other aggressive malignancies arising outside of the central nervous system (CNS). In contrast, this benefit has yet to translate into patients with GBM in phase III clinical trials to-date (Bristol-Myers Squibb, 2017; Filley et al., 2017).

Age is one of the primary risk factors for cancer, with individuals ≥65 years of age accounting for 60% of newly diagnosed malignancies and 70% of all cancer-related deaths (Ries et al., 2006). A similar report highlighted an age adjusted cancer mortality rate for persons ≥65 at ∼16 times higher than the mortality rate for those <65 (Berger et al., 2006). Care for these individuals is challenging due to the number of diseases elderly subjects are at high risk for, which also raises the likelihood of presenting multiple comorbidities during advanced aging (Yancik et al., 1998, 2001). Similarly, the incidence and mortality rate of GBM increases during advanced aging with a median diagnosis at 64 years old (Young et al., 2017). Aging is a complex process that affects nearly all aspects of the immune system (Nikolich-Zugich, 2018). In general, advanced aging decreases immune system effectiveness, as is evidenced in elderly individuals who receive the influenza vaccine (McElhaney and Dutz, 2008). Aging also negatively impacts apoptotic cell clearance (Aprahamian et al., 2008), the numbers of naïve T cells (Cambier, 2005), and the wound healing response (Wick et al., 2010). T cell senescence increases with progressive aging through the induction of p16 (Liu et al., 2009). Aging also affects T cell receptor (TCR) signaling in CD4^+^ T cells, due in part to decreased miR-181a; a microRNA highly expressed in normal T cells (Adachi and Davis, 2011; Li et al., 2012; Chen et al., 2013). Both aged mice (Rossi et al., 2005) and human (Pang et al., 2011) hematopoietic stem cells (HSC) possess a myeloid-biased differentiation potential as compared with HSC from young subjects. Moreover, macrophages from donor subjects with advanced age possess decreased capacity for antigen presentation as compared to young donors (Davila et al., 1990; Gon et al., 1996). Neuro-immune interactions are also affected by aging in the brain (Carson et al., 2006) that include a significant upregulation of MHCII and CD11b on microglia (Rogers et al., 1988; Perry et al., 1993), as well as an accumulation of brain-resident dendritic cells (Bulloch et al., 2008; D’Agostino et al., 2012; Kaunzner et al., 2012). However, it is not yet clear as to whether aging possesses a distinct impact on immunosuppressive gene expression across select tissues that contribute to a microenvironment permissive for oncogenesis, tumor progression, and/or resistance to immunoregulatory-based therapies.

Our laboratory previously demonstrated a negative impact of advanced age on the survival of animals engrafted with syngeneic experimental brain tumors (Ladomersky et al., 2016). C57BL/6 mice intracranially injected with murine GL261 glioma cells at 72–74 weeks old, which is similar in humans to a time frame associated with the median age of a GBM patient diagnosis (Fox et al., 2007; Dutta and Sengupta, 2016), have a decreased mOS as compared to animal subjects with an age of 6–8 weeks old (27.5 and 21.5 days, respectively, *P* = 0.029, *n* = 10–12/group); the latter of which is similar in age to a human teenager. The negative impact of advanced aging was coincident with increased immunosuppressive IDO1 gene expression in the normal, non-malignant mouse brain. More recently, we discovered that a substantial proportion of C57BL/6 mice intracranially injected with GL261 at 6–8 weeks of age experience long-term survival when simultaneously treated with radiation (RT), anti-PD-1 mAb, and IDO1 enzyme inhibitor (Ladomersky et al., 2018). The brain tumor survival benefit provided by this treatment, however, was negatively affected by animal subjects with advanced age as compared with young subjects (Ladomersky et al., 2018). Importantly, there was no significant difference in tumor infiltrating leukocyte populations between the young and aged subjects within treatment groups. To our knowledge, this is the first preclinical primary brain cancer study to demonstrate a negative impact of aging on survival after treatment with immunotherapy.

Further supporting the hypothesis that, advanced aging mediates suppression of immune system efficacy against a tumor challenge event, previous work showed that splenocytes isolated from young, but not old immunized subjects, were able to eradicate subcutaneous tumors in mice (Schreiber et al., 2012). Specifically, immunodeficient recombination activating gene knockout mice (Rag^−/−^) were subcutaneously engrafted 8101 cells arising from mice treated with UV-irradiation, and possessing a somatic mutation in the T cell-recognized antigen RNA helicase, p68. Splenocytes isolated from 5 month old mice and immunized with live 8101 cells, but not those from immunized 29 month old mice, eradicated 8101 cell-based tumors post-adoptive transfer into Rag^−/−^ mice. Interestingly, melanoma patients ≥62 years of age show increased responsiveness to anti-PD-1 mAb treatment as compared with younger human subjects (Kugel et al., 2018). Recapitulating this clinical observation, 10 month old animal subjects, which roughly correlate to the human age of 38–47 years and engrafted with murine BSC9AJ2 melanoma cells, show decreased tumor growth as compared to 2 month old engrafted mice after treatment with anti-PD-1 mAb (Kugel et al., 2018). This highlights an interesting dichotomy suggesting that, the productivity of an anti-tumor immune response during treatment with immunotherapy likely depends on both the cancer type and age of the host. These combined findings may suggest that GBM is an outlier when considering its place in cancer immunology and immunotherapy. Accordingly, we previously found an inverse association between high CD3ε and CD8α gene expression with GBM patient survival (Zhai et al., 2017), which is a diametrically opposite finding as compared with non-small cell lung cancer and melanoma (Zeng et al., 2016; Zhai et al., 2018).

In our current study, we explored the associations between human: (i) aging; (ii) levels of gene expression associated with immunoregulation inside the brain; (iii) immunological changes in the peripheral blood; and (iv) incidence and mortality of patients with primary brain and other CNS tumors. Epidemiological analyses of GBM patient characteristics were compared across the Surveillance, Epidemiology, and End Results (SEER) database, age-dependent gene expression levels of normal human brain from the GTEx database, and age-associated changes in normal human peripheral blood leukocytes from the 10k Immunomes database. Our study confirms the striking observation that the brain cancer mortality rate is actively rising, with a particular enrichment among the elderly population in the United States. The incidence of GBM is 3.4× higher among individuals ≥65 years old, as compared to those <65, while the mortality rate for individuals ≥65 years old with GBM is 7× higher as compared to GBM patients <65. Gene expression levels of immunosuppressive IDO1 increased in the normal human brain and was maximal among individuals aged 60–69 years old. As compared to younger subjects, there was a maximal incidence of circulating immunosuppressive regulatory T cells (Tregs) and a significantly decreased cytolytic CD8^+^ T cell population among the 60–69 year old age group. Our study found a cumulative peak index for immunosuppressive and/or immunoregulatory mediators during the time frame associated with the median age of a GBM patient diagnosis. These data raise the intriguing possibility that aging suppresses mechanisms of immunosurveillance and responsiveness to immunotherapy, which is associated with the increasing number of elderly patients with brain cancer and the failure of immunotherapy to benefit GBM patients in phase III clinical trials to-date, respectively.

## Materials and Methods

### Data

#### Life Expectancy Data

Life expectancy data were analyzed from the Center for Disease Control and Prevention. Data were accessed through the National Center for Health Statistics portal^1^. The filename used was *Life expectancy at birth and at age 65, and at age 75, by sex, race, and Hispanic origin: United States, selected years 1900–2016*.

#### Surveillance of Epidemiology and End Results Database (SEER)

All population, incidence, and mortality data from the SEER database were accessed through SEER^∗^Stat (Version 8.3.5^2^). Population-level data were accessed through a Frequency Session. Variables examined were: (1) Age recode with <1 year olds; and (2) year. Incidence and mortality data were accessed through a Rate Session. Variables examined for incidence include: (1) age recode with <1 year olds; (2) year of diagnosis; (3) histology recode – broad groupings; (4) histology recode – brain groupings; and (5) COD to site recode. The COD to site recode was used to analyze the mortality rate of GBM. Variables examined for mortality include: (1) age recode with <1 year old; (2) year of death; and (3) cause of death recode. All rate data were crude/non-age-adjusted. Data were accessed on 12-05-2018.

#### NCI 2018 Estimates

Estimated incidence and mortality data were retrieved from the NCI Cancer Stat Facts website^3^. The top 16 causes of cancer were analyzed for both incidence and mortality. Comparison across the 16 cancers for the mortality to incidence ratio estimate were calculated by dividing the estimated mortality with the estimated incidence in the year 2018. Cost data were accessed through the NCI Cancer Prevalence and Cost of Care Projections website^4^. Cost per patient was calculated by taking the 2018 estimated total charges for each type of cancer and dividing it by the 2018 estimated incidence of each cancer type.

#### TCGA

Survival data for GBM patients were analyzed from the cancer genome atlas. The data were accessed using the UCSC Xena portal. Data were accessed on 02-01-2019.

#### 10k Immunomes

Normal human peripheral blood cell data were analyzed from the 10k Immunomes database. The data were accessed through the UCSF portal^5^. All data analyzed were CyTOF data plotted by age. Data were accessed on 06-22-2018.

#### GTEx

Gene expression data for normal human tissues were analyzed from the GTEx database. Data were accessed through the dbGaP portal. All data were represented as log-transformed fragments per kilobase of transcript per million mapped reads (FPKM). These data were then plotted by subject age.

### Statistical Analysis

10k Immunomes and TCGA expression data are represented as the mean ± SEM. GTEx data are represented by each individual value. The statistical significance of differences in cell counts (CyTOF) and gene expression (log-transformed FPKM) between two groups was determined by Student *t*-test. Differences among multiple groups were assessed using ANOVA with *post-hoc* Tukey test. The statistical significance of TCGA survival data was determined by Log-rank test. Data were analyzed using Prism software (GraphPad Software). A *P*-value less than 0.05 was considered significant.

## Results

### The Rate of Cancer Incidence Increases With Age

**FIGURE 1:**
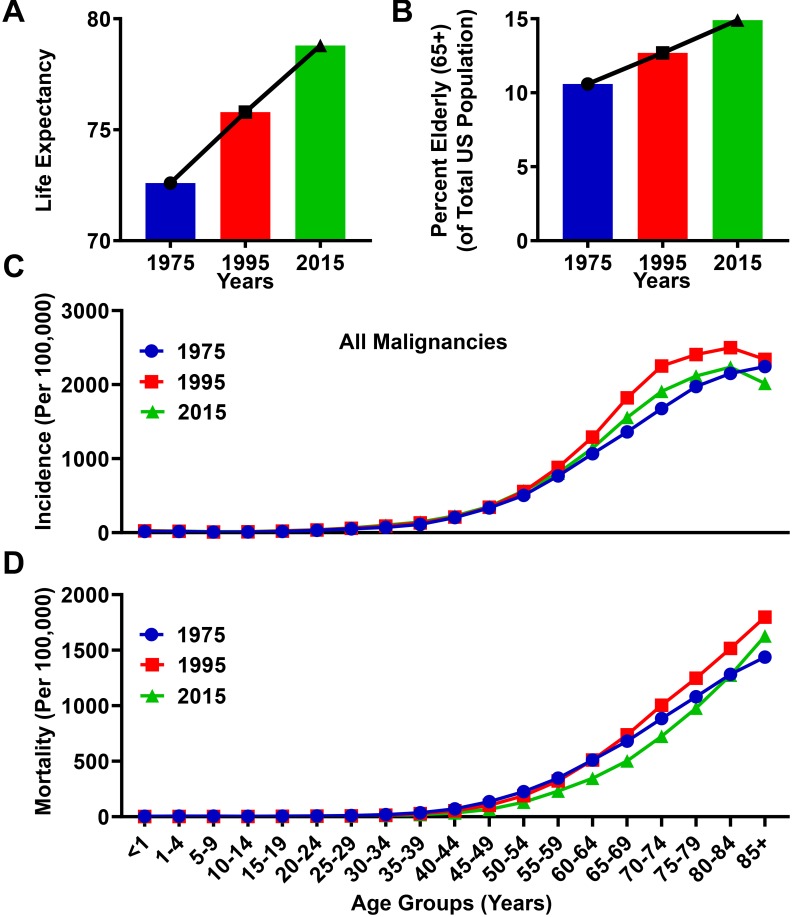
Progressive aging is associated with an increased rate of cancer incidence and mortality. **(A)** Life expectancy from the Center for Disease Control and Prevention (CDC) database for an average lifespan in the United States between the years 1975 and 2015. **(B)** Population data from the Surveillance, Epidemiology, and Ends Results (SEER) database representing the total number of elderly human subjects (ages ≥65) divided by the total population in the United States for the years 1975, 1995, and 2015. **(C)** Incidence and **(D)** mortality rates from the SEER database for all malignancies in the years 1975, 1995, and 2015. Rates are defined as number of cases and deaths, respectively, divided by the population in each age category and multiplied by 100,000 people (per 100,000).

Over the past 40 years in the United States, improved healthcare systems and enhanced awareness of proper nutrition and exercise have led to substantial increases in mean life expectancy, rising from 72.6 years of age in 1975, to 78.8 years of age in 2015 (Figure 1A). Coincident with the increasing life expectancy in the United States, the proportion of elderly (≥65 years) individuals is rising. The elderly age group represented 10.6% of the total population in 1975, and increased to 14.9% in 2015 (Figure 1B). Both the incidence and mortality rates associated with cancer diagnoses are higher among the elderly population as compared to individuals <65 years of age (Figure 1C,D). In the year 1975, the incidence and mortality rate within the elderly population for all malignancies was 1,732/100,000 people and 942/100,000, respectively, whereas in the year 2015, the incidence rate for all malignancies in the elderly population was 1,876/100,000 and the mortality rate was 879/100,000. Compared to the population <65, these incidence and mortality rates were 9.6 and 12.1 times higher, respectively, in 1975, and 7.1 and 13.6 times higher, respectively, in 2015. The slight decrease in mortality rate between 1975 and 2015 is most likely due to improved detection and treatment techniques in select cancers. However, due to the increasing elderly population, the absolute numbers of cancer related mortalities in this population has risen from 214,173 in 1975, to 419,389 in 2015. Together, these data suggest an association between the aging population and an absolute increase of cancer-related deaths in the elderly population of the United States.

### Brain Cancer Diagnoses Pose a Growing Challenge to the United States Healthcare System

**FIGURE 2:**
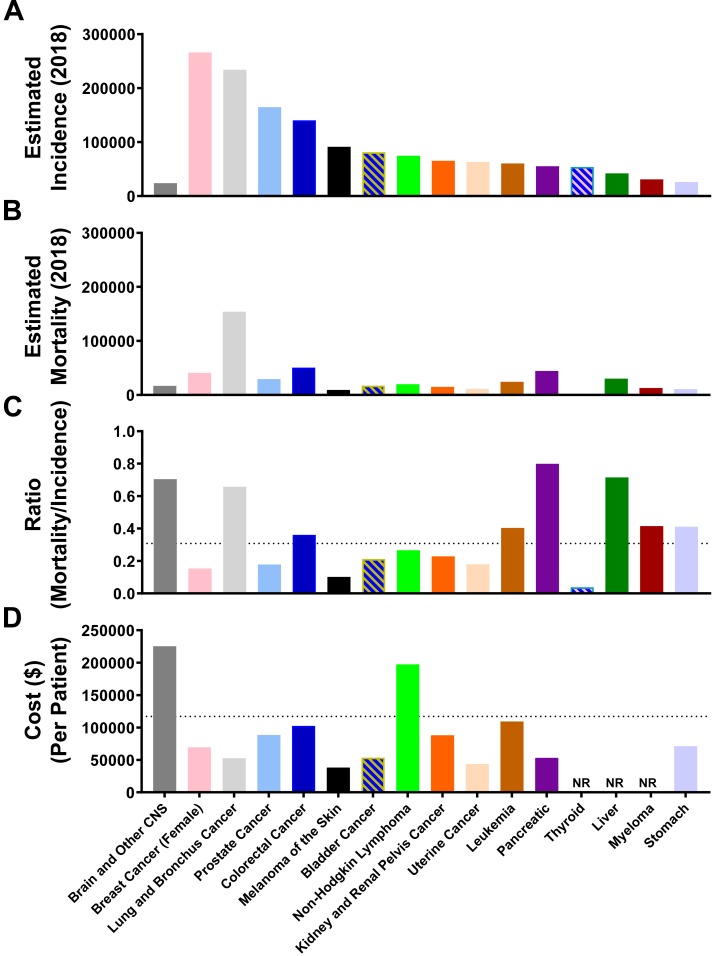
Brain and other CNS cancer is associated with a high mortality/incidence ratio and is expensive to treat. The estimated total **(A)** incidence and **(B)** mortality rate for the top 16 cancer types according to NCI Cancer Stat Facts. **(C)** The calculated mortality to incidence ratio (estimated mortality/estimated incidence) for the top 16 cancer sites according to NCI Cancer Stat Facts. The dotted line represents an average across all 16 cancer types. **(D)** Cost (United States dollars) per patient for each of the top 16 cancer types. Dotted line represents the average for all 16 cancer types. NR, Not reported.

Primary brain tumors arising from a transformed cell within the CNS is a relatively rare form of cancer, with the 16th highest rate of incidence among all cancers, and an estimated 23,880 new patient diagnoses in 2018 (Figure 2A). Dwarfing this number is the estimated 266,120 new diagnoses for patients with breast cancer in the year 2018. The overall ratio of estimated breast cancer incidence, as compared with brain cancer, is 11:1. In contrast, the estimated mortality rate for patients diagnosed with CNS cancer is 16,830 in 2018 (Figure 2B), and is 40,920 for individuals diagnosed with breast cancer. The overall ratio of breast cancer-associated mortalities, as compared to mortality due to CNS cancer, is only 2.4:1. Aggressiveness of cancer diagnoses can also be calculated with the mortality-to-incidence ratio (MIR), which is determined by dividing overall mortality with the number of new diagnoses in a given year (Choi et al., 2017). CNS cancer as a group possesses the third highest MIR of 0.70, which is only exceeded by pancreatic and liver cancer, with MIR scores of 0.80 and 0.72, respectively (Figure 2C). Leading all other groups, treatment for CNS cancers is the most costly on a per patient basis, with an average cost estimated to be $225,364 in 2018 (Figure 2D).

### Brain Cancer Incidence/Mortality Is Rising and Enriched in the Elderly

**FIGURE 3:**
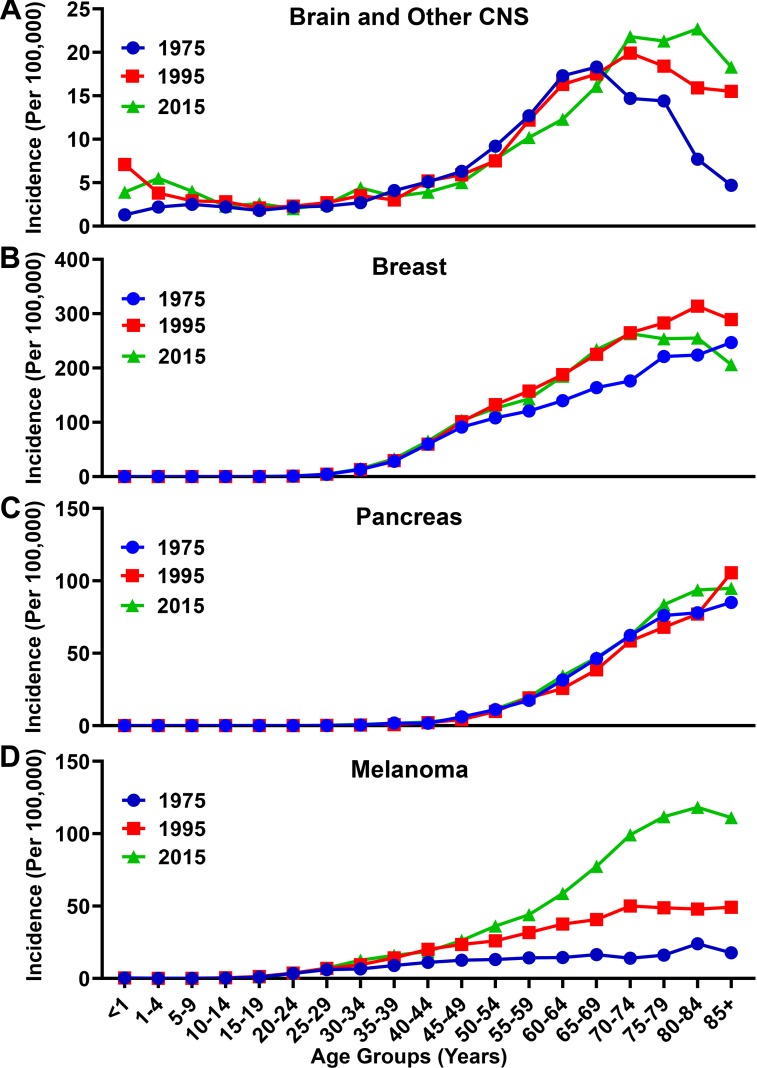
Incidence rate for brain, breast, pancreatic, and melanoma malignancies over time. Incidence rates for **(A)** brain and other CNS, **(B)** breast, **(C)** pancreatic, and **(D)** melanoma malignancies analyzed from the SEER database for the years 1975, 1995, and 2015. Rates defined as number of cases divided by the population in each age category multiplied by 100,000 people (per 100,000).

Although there has only been a small change in the incidence and mortality rates of all malignancies (Figure 1C,D) from 1975 to 2015, analysis of incidence data for brain and other CNS cancer shows a different trend (Figure 3A). The incidence rate has increased overall from 5.4/100,000 in 1975, to 7.0/100,000 in 2015. The overall increase, however, is primarily attributable to the elderly population. In 1975 the incidence rate for individuals with brain and other CNS malignancies ≥65 years of age was 14.2/100,000, which increased nearly 37% to 19.4/100,000 in 2015. In that same time span, the incidence rate for those <65 years of age only slightly increased from 4.4/100,000 in 1975 to 5.0/100,000 in 2015. When comparing other malignancy types including breast, pancreatic, and lung, a similar trend of increased incidence was observed. Breast cancer incidence rates increased from 47.4/100,000 to 78.0/100,000 in the total population over the same time span, and from 192.4/100,000 to 242.6/100,000 among the elderly population (Figure 3B). Pancreatic cancer incidence rates rose more slightly, from 9.4/100,000 to 14.5/100,000 in the total population and from 63.3/100,000 to 68.8/100,000 among the elderly over the past 40 years (Figure 3C). The incidence of melanoma increased from 6.8/100,000 to 28.4/100,000 in the total population and from 16.7/100,000 to 97.7/100,000 within the elderly population from 1975 to 2015 (Figure 3D). Overall, more people were diagnosed with malignancies in 2015 as compared with 1975.

**FIGURE 4:**
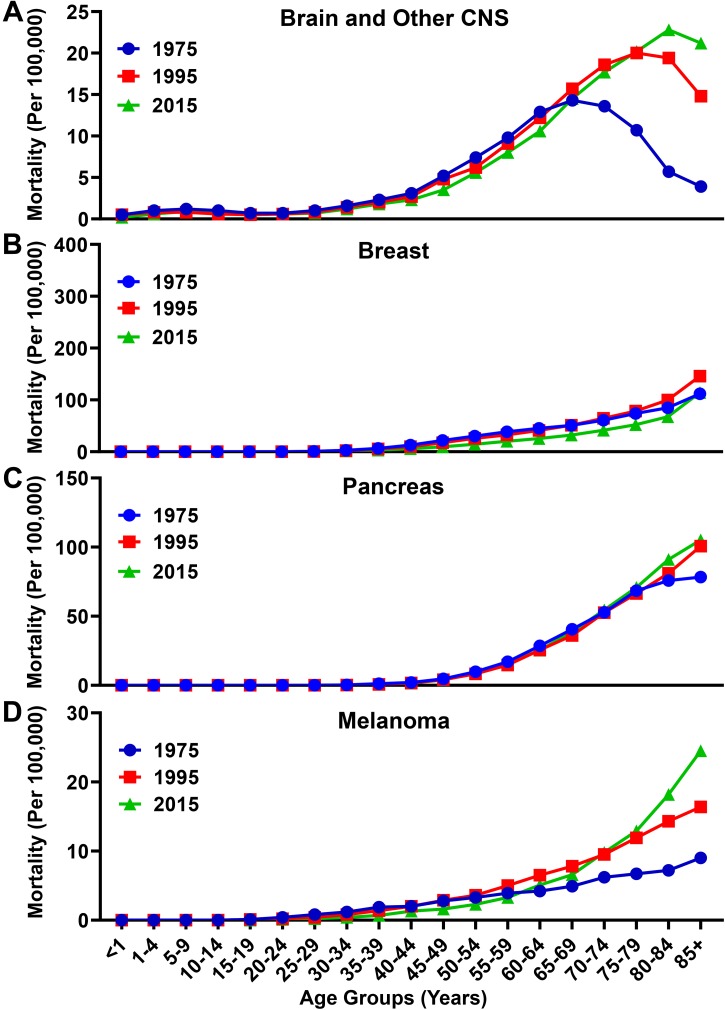
Mortality rate for brain, breast, pancreatic, and melanoma malignancies over time. Mortality rates for **(A)** brain and other CNS, **(B)** breast, **(C)** pancreatic, and **(D)** melanoma malignancies analyzed from the SEER database for the years 1975, 1995, and 2015. Rates defined as number of deaths divided by the population in each age category multiplied by 100,000 people (per 100,000).

Similar to the increased rate of incidence, the overall mortality rate among individuals with brain and other CNS cancers increased in the total population from 3.8/100,000 in 1975 to 5.1/100,000 in 2015 (Figure 4A) The mortality rate for individuals with brain and other CNS malignancies ≥65 years old was 11.6/100,000 in 1975, and increased by 56% to 18.1/100,000 in 2015. In contrast, the mortality rate for individuals <65 years of age rose slightly from 2.8/100,000 in 1975 to 2.9/100,000 in 2015. The overall mortality rate for individuals with breast cancer decreased from 15.1/100,000 in 1975 to 13.1/100,000 in 2015, and from 66.8/100,000 to 52.9/100,000 among individuals ≥65 years of age during the same time periods, respectively (Figure 4B). The overall mortality rate for individuals with pancreatic cancer rose from 9.0/100,000 to 13/100,000 from 1975 to 2015, but only increased slightly from 56.2/100,000 to 63.0/100,000 among individuals ≥65 years of age during the same time periods, respectively (Figure 4C). The overall mortality rate increased for patients with melanoma from 1.8/100,000 to 2.8/100,000 from 1975 to 2015 and increased from 6.2/100,000 in 1975 to 12.2 in 2015 among individuals ≥65 years of age during the same time periods, respectively (Figure 4D).

**FIGURE 5:**
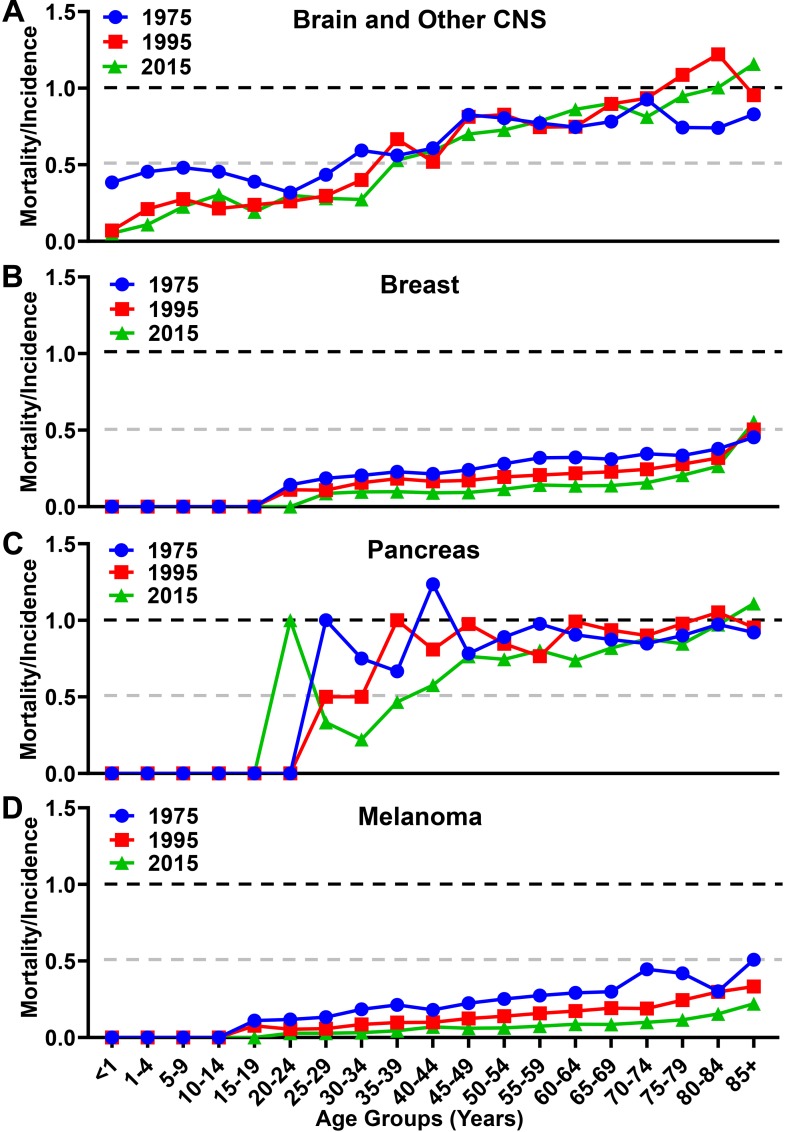
Mortality to incidence ratio for brain, breast, pancreatic, and melanoma malignancies over time. The calculated mortality to incidence ratio of **(A)** brain and other CNS, **(B)** breast, **(C)** pancreatic, and **(D)** melanoma malignancies for each year calculated from SEER incidence and mortality data. Instances where the ratio is over 1.0 are due to the lag time between diagnosis and mortality in GBM and pancreatic cancer.

When analyzing the MIR as a function of aging, there were differences in trends among the malignancies analyzed. While the overall MIR for individuals with brain cancer increased slightly from 0.70 in 1975 to 0.73 in 2015 (Figure 5A), there is a larger increase from 0.82 in 1975 to 0.93 in 2015 among individuals ≥65. In contrast, the overall MIR for individuals with breast cancer decreased over the same time span from 0.32 in 1975 to 0.17 in 2015 (Figure 5B), with a similar decrease of 0.35 in 1975 to 0.22 in 2015 among individuals ≥65. The overall MIR for individuals with pancreatic cancer was 0.96 in 1975 and slightly decreased to 0.90 in 2015 (Figure 5C), whereas this figure is relatively unchanged within the elderly population at 0.89 in 1975 to 0.90 in 2015. The overall MIR for individuals with melanoma decreased from 0.26 to 0.10 in 2015, and from 0.37 to 0.12 among individuals ≥65, respectively (Figure 5D). Taken together, these data show substantially different trends of aggressiveness, historically and currently, with brain cancer and other CNS cancers demonstrating a substantial increase in overall incidence, mortality, and MIR among the elderly portion of the United States population.

### The Incidence and Mortality of Individuals With GBM Is Progressively Enriched With Aging

**FIGURE 6:**
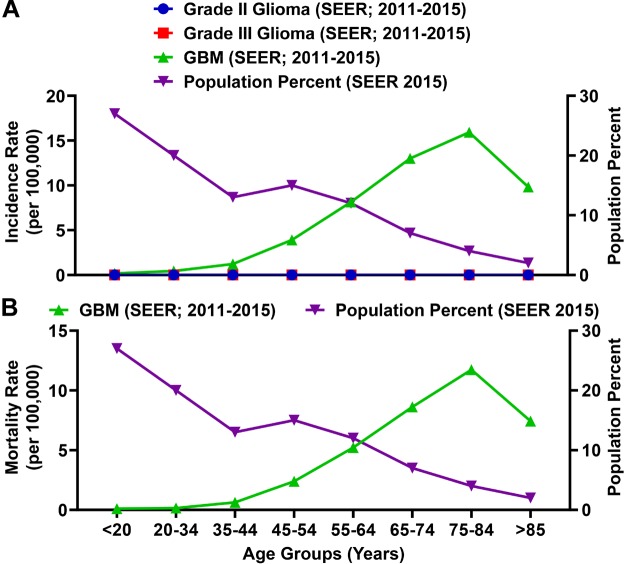
The incidence and mortality rates for glioblastoma (GBM) patients are enriched among the elderly population. **(A)** SEER incidence rates (per 100,000) for grade II glioma, grade III glioma, and GBM (grade IV glioma; all left axis) compared to the percent of the total population represented for each age group. Grade II and III glioma incidence rates calculated by SEER were >0.015 (per 100,000) for each age category. **(B)** The SEER mortality rate for GBM as compared to the percent of the total population represented for each age group. Incidence and mortality data is represented as a summation for the years 2011–2015.

Glioblastoma (GBM; grade IV) is the most common primary malignant brain tumor in adults and accounts for 54% of malignant glioma diagnoses (Wen and Kesari, 2008). Among total glioma incidence, the rate of a GBM diagnosis is substantially higher as compared with grade II and grade III glioma (Figure 6A). The rate of a GBM patient diagnosis and mortality is enriched among the elderly population (Figure 6) and the MIR for individuals ≥65 years of age is 1.00.

**FIGURE 7:**
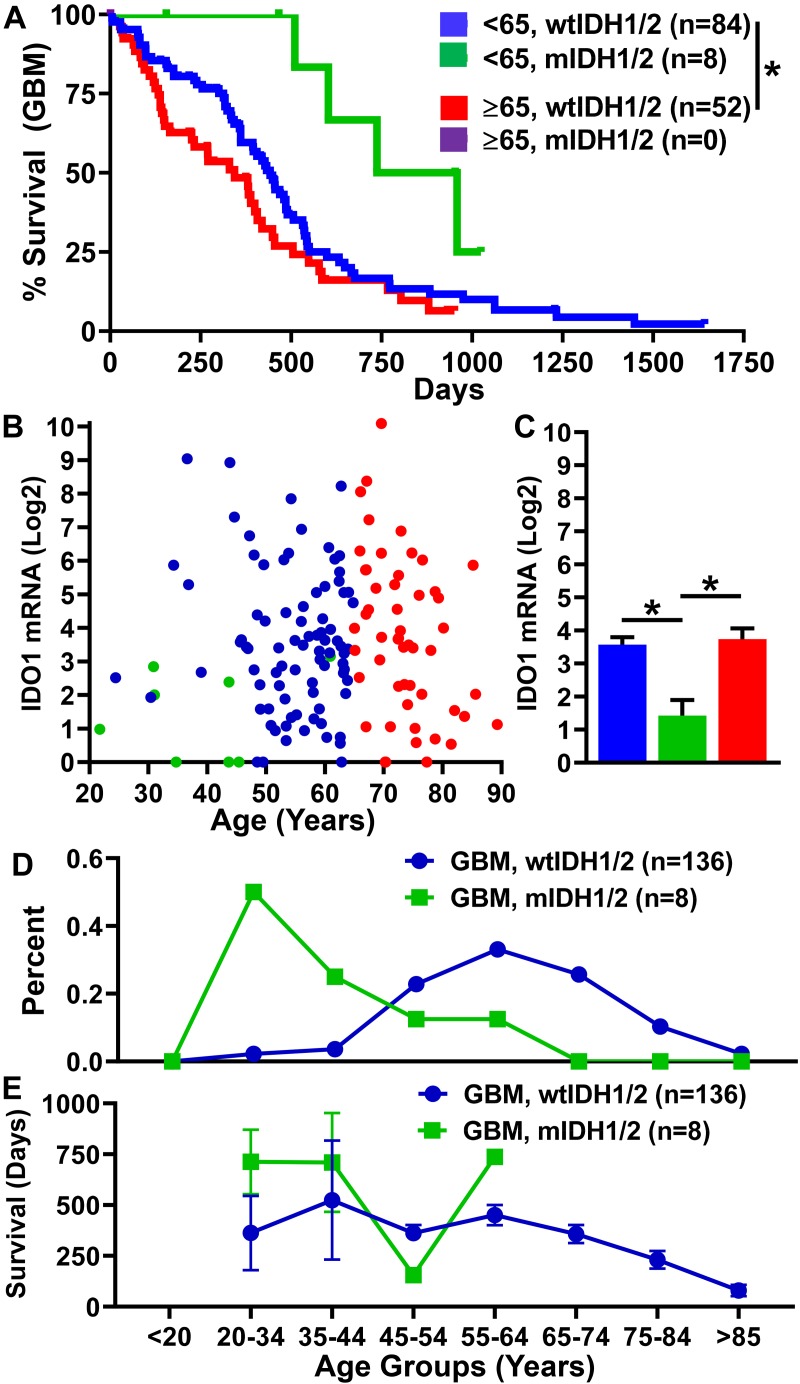
Prognosis is worse for individuals with GBM ≥65. Analysis of GBM data for individuals with corresponding patient data, gene expression (HiSeq) data, and a reported mIDH1/2 status from the TCGA. **(A)** Survival data of individuals with wild-type IDH1/2 <65 (*n* = 84), ≥65 (*n* = 52), and those with mIDH1/2 (*n* = 8). IDO1 gene expression **(B)** dot plot and **(C)** bar graph of individuals with wild-type IDH1/2 <65 (blue), ≥65 (red), and those with mIDH1/2 (green). **(D)** The percentage of GBM diagnoses within each age category for individuals with wtIDH1/2 (blue) and mIDH1/2 (green). **(E)** Survival time in days of individuals diagnosed in each age category. ^∗^*P* < 0.05.

Not only is GBM enriched among the elderly population, but the prognosis for these patients is substantially worse as compared with individuals <65 years of age. We analyzed overall survival and expression data from the cancer genome atlas (TCGA) for individuals with GBM who possessed corresponding patient data, expression data, and a reported IDH status (*n* = 144). There were only 8 patients within the dataset reporting the presence of mutant IDH (mIDH), all of which were <65 years of age. There is a significantly shorter mOS of 11.3 months among individuals ≥65 years of age as compared with individuals <65 years of age (14.5 months; *P* = 0.019) (Figure 7A). In contrast, mOS for the eight individuals with mIDH GBM is a striking 27.9 months. Intratumoral gene expression levels for the immunosuppressive enzyme, IDO1, is similar for individuals <65 and ≥65 with wild-type IDH status, and significantly decreased among mIDH GBM (Figure 7B,C) as was previously reported (Zhai et al., 2017). While a majority of individuals with wild-type IDH GBM were diagnosed later in life, 50 percent of individuals with mIDH GBM were diagnosed before the age of 35 (Figure 7D). Not surprisingly, overall survival time decreased with progressively increasing age among individuals with GBM (Figure 7E). Taken together, the data indicate that individuals with a wild-type IDH GBM, which constitute the majority of new diagnoses, possess substantial comorbidity with advanced aging.

### The Median Age of a GBM Patient Diagnosis Is Coincident With Enhanced Global Immunosuppression

**FIGURE 8:**
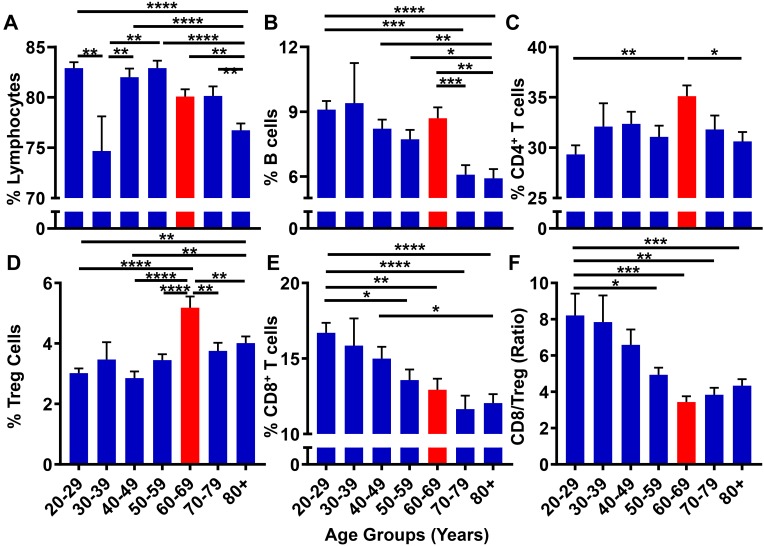
The peripheral blood CD8^+^ T/Treg ratio decreases with progressive aging in healthy human subjects. 10k immunomes CyTOF data from the peripheral blood of healthy patients analyzed for **(A)** total lymphocytes, **(B)** B cells, **(C)** CD4^+^ T cells, **(D)** CD8^+^ T cells, **(E)** Tregs (CD4^+^CD25^+^FoxP3^+^), and **(F)** CD8^+^ T/Treg ratio. Data was analyzed among age groups including 20–29 (*n* = 110), 30–39 (*n* = 14), 40–49 (*n* = 76), 50–59 (*n* = 78), 60–69 (*n* = 72), 70–79 (*n* = 57), 80+ (*n* = 127). ^∗^*P* < 0.05; ^∗∗^*P* < 0.01; ^∗∗∗^*P* < 0.001; ^∗∗∗∗^*P* < 0.0001.

Although GBM is generally considered as a disease initiated by genetic mutations, it may also arise due to immunological dysfunction (Shurin, 2012); although it is not clear which event(s) precede(s) the other in contributing to GBM cell initiation/outgrowth. While immunosuppression is routinely analyzed in GBM patients following a diagnosis (Beatty and Gladney, 2015), few studies have prospectively investigated the correlation between progressive aging and the level of immunosuppression in human subjects. With the hypothesis that the suppression of the normal immune system contributes to the microenvironment necessary to facilitate GBM cell initiation, the 10k Immunomes database was profiled for immune system status among healthy human subjects. There were substantial changes in the peripheral immune system associated with aging. While there were no noticeable trends among total lymphocytes (Figure 8A) and CD19^+^ B cell (Figure 8B) levels, analysis of T cell subpopulations revealed an interesting phenotypic trend. As demonstrated in Figure 8C, CD4^+^ T cell levels are maximal in the 60–69 age range. Drilling down into the CD4^+^ T cell subset revealed that the maximal increase was primarily attributable to the increase of immunosuppressive regulatory T cells (Tregs; CD3^+^CD4^+^FoxP3^+^) within the same 60–69 age group (Figure 8D). Strikingly, the cytotoxic CD8^+^ T cells coincidently decreased with progressive aging (Figure 8E). Since a high CD8^+^ T/Treg ratio is associated with improved overall survival, (Yue et al., 2014; Shang et al., 2015) and because there is a decreased CD8^+^ T/Treg ratio during advanced aging of normal human healthy individuals that is maximal in the 60–69 age group, the data collectively demonstrate a trend for increased immunosuppression in the peripheral blood coincident with the median age of a GBM patient diagnosis.

**FIGURE 9:**
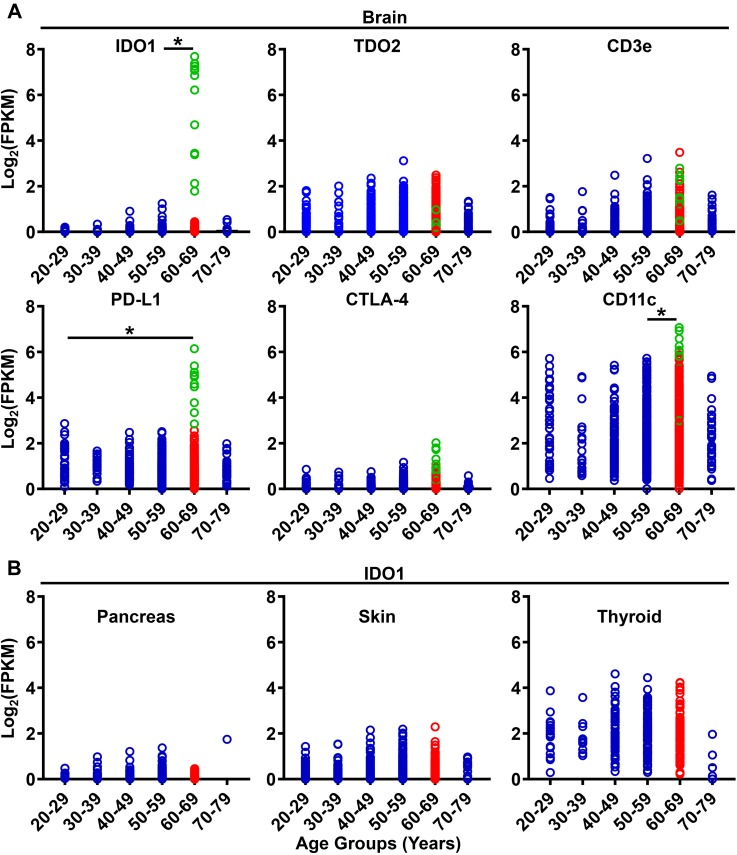
Gene expression for immunosuppressive and immunoregulatory factors increase in the normal human brain with aging. **(A)** GTEx gene expression analysis of IDO1, TDO2, CD3ε, PD-L1, CTLA-4, and CD11c in the brain. Data was analyzed among age groups including 20–29 (*n* = 38), 30–39 (*n* = 19), 40–49 (*n* = 123), 50–59 (*n* = 386), 60–69 (*n* = 549), 70–79 (*n* = 37). Green circles across gene panels share the same sample IDs. **(B)** GTEx gene expression analysis of IDO1 in the normal human pancreas (*n* = 167), skin (*n* = 812), and thyroid (*n* = 280). ^∗^*P* < 0.05.

We previously demonstrated that immunosuppressive IDO1 is significantly increased in the normal healthy brain of 72–74 week old mice as compared with young 6–8 week old counterparts; independent of tumor burden (Ladomersky et al., 2016, 2018). To determine whether this observation is generalizable to humans, RNA-sequencing data from the GTEx database was analyzed for mRNA expression levels of established immunoregulatory genes in normal human brain (Figure 9A). Similar to our analysis of increased IDO1 expression levels in the brain of mice during advanced aging, IDO1 is also increased in the normal human brain among the 60–69 age group as compared to human subjects in the 50–59 age group (*P* = 0.02; Figure 9A; Supplementary Figure S1). Although this trend remained when comparing the 60–69 age group to other age cohorts, it did not achieve statistical significance due to insufficient human samples for comparison. Similar to increased IDO1 mRNA expression, gene expression for immunosuppressive PD-L1 was also highest in the 60–69 year old age group as compared with the 20–29 year old age group (*P* = 0.01). Unexpectedly, CD11c mRNA levels, a marker traditionally associated with immune sentinel dendritic cells, was also increased in the 60–69 age group as compared with human subjects in the 50–59 age group (*P* = 0.02; Figure 9A). Interestingly, samples containing the highest IDO1 gene expression (Figure 9A; green) also showed high PD-L1 and CD11c levels, possibly suggesting an immunosuppressive phenotype with dendritic cell accumulation in those samples. In contrast to the enrichment of select immunosuppressive factors significantly increased in the normal human brain, IDO1 mRNA levels do not significantly change in pancreatic, skin, or thyroid tissues across age groups (Figure 9B; Supplementary Figure S2), nor any other human tissue analyzable in the GTEx database. Collectively, these data confirm that systemic and brain-specific immunoregulatory factors favoring immunosuppression are maximal during the time frame associated with the median age of a GBM patient diagnosis.

## Discussion

The Director of the National Cancer Institute, Dr. Normal E. Sharpless, M.D., formerly an investigator within the National Institute of Aging, has highlighted the importance of the relationship between cancer and aging (Sharpless, 2018). Cancer is a disease enriched among the elderly and is often associated with deficits in normal immunosurveillance (Dunn et al., 2004; Zhong et al., 2016). Aging is associated with progressive immunological changes throughout the body, including involution of the thymus, a critical site for pre-T cell education and development into mature naïve T cells, a shift of the circulating T cell repertoire from a naïve to memory phenotype, an absolute decrease in the number of naïve T and B cells, a reduction in cytokine signaling, and reduced uptake of antigens and/or microbes by dendritic cells. To further understand the immunological changes that occur during aging, and whether these differences provide for preventative or therapeutic applications to individuals that will be diagnosed with malignant primary brain cancer, we comprehensively studied multiple bioinformatic database repositories for aging-related associations between GBM patient incidence and mortality, changes in the peripheral immune response of normal healthy human subjects, and gene expression throughout the normal healthy human body for immunoregulatory factors. Accordingly, the scientific premise of our investigation aimed to identify meaningful relationships between the ages of a GBM patient diagnosis, with differences in the immune system that are potentially therapeutically targetable.

During our investigation, we discovered that the United States rate of incidence and mortality for brain and other CNS malignancies has been rising over the past 40 years, and due to its enrichment among the elderly population, may reflect the increased number of individuals that have a higher propensity for brain cancer diagnoses as compared with younger subjects (Figure 1, 3, 4, 5). This differs from the trend for all malignancies, where the incidence and mortality rates decline between the years 1995 and 2015 (Figure 1C,D). This may be due to an increase in the number of effective therapies for treating other types of cancer, as well as improved prevention and detection techniques for high incidence malignancies including breast cancer and melanoma. According to a 2018 projection by the NCI (see text footnote 3), brain and other CNS malignancies have the third highest mortality:incidence (MIR) ratio when comparing across major cancer subtypes, which reflects a high degree of aggressiveness. Strikingly, brain cancer and other CNS malignancies were predicted to be the most expensive cancer on a per patient basis in the year 2018 (Figure 2). Further investigation of glioma incidence data revealed that GBM diagnoses are highly enriched among the elderly. When the mortality data was accounted for, the MIR for individuals with GBM between the years 2011–2015 was 1.00 (Figure 6). Analysis of TCGA data revealed that not only are the elderly at a higher risk for GBM, but this cohort of individuals also have a significantly worse prognosis when diagnosed with GBM (Figure 7). Further evaluation revealed a peak GBM patient incidence/mortality rate that corresponded to the maximal levels of immunosuppressive IDO1 and PD-L1 mRNA expression in the CNS, as well as immunosuppressive peripheral Treg abundance (Figure 8, 9). Interestingly, the CD4^+^FoxP3^−^ T cells were less affected by aging (Figure 8). A hypothesis for the increased immunosuppressive markers in the 60–69 age group, but not among even older age groups, is that only a subset of individuals experience this increase. It’s also possible that the transient increase in local immunosuppression synergizes with peripheral mechanisms during a timeframe of substantial hormonal imbalance (i.e., menopause). Interestingly, CD11c mRNA expression also increased in the elderly human brain, which may be associated with the accumulation of brain-resident cells (Bulloch et al., 2008; D’Agostino et al., 2012; Kaunzner et al., 2012).

Since cancer is enriched among the elderly, combined with the increasing size of the population with advanced age (Figure 1A,B), it was surprising to find that not all malignancies had an associated increased mortality rate when comparing data between 1975 and 2015. Several hypotheses may explain the rise in mortality of primary malignant brain cancer as compared to other malignancies including: (i) the lack of effective treatment options for patients diagnosed with incurable brain tumors as compared to therapeutic improvements for non-CNS malignancies; (ii) a steady enrichment in factors that promote the development of more malignant primary brain tumors; (iii) a more aggressive natural history in established primary brain tumors; (iv) an absolute increase in the number of elderly individuals that have a higher chance for developing primary malignant glioma; (v) an increase over time of elderly patients diagnosed with malignant brain tumors due to more aggressive patient work ups providing for a larger pool of individuals contributing to the mortality statistics; or (vi) a combination of all stated potential conferring factors.

The CNS is a potently immunosuppressive and immunospecialized organ, as compared with peripheral tissues (Carson et al., 2006). Through normal human subject gene expression analysis, we found increased immunosuppressive IDO1 in the brain that was maximally enhanced in the 60–69 year old subgroup, which is complementary to our previous work demonstrating increased IDO1 in the normal brain of mice during advanced aging (Ladomersky et al., 2016, 2018). Unexpectedly, IDO1 enzyme activity was not increased in the aged brain, questioning the functionality of the increased brain IDO1 expression. Recent studies in our laboratory and others have hypothesized that the immunosuppressive function of IDO1 may be, in-part, independent of its associated enzyme activity (Pallotta et al., 2011; Wainwright et al., 2012; Zhai et al., 2017). Although additional studies are required to fully understand the relationship between increased human brain IDO1 expression and advanced aging, a hypothesis that currently fits the available data is that IDO1 expression increases CNS immunosuppression through non-enzyme activity. Whether the basal increase of brain IDO1 expression contributes to the microenvironment required for GBM cell initiation is also an intriguing consideration.

Another factor for considering the effects of aging on immunosuppression and GBM onset is the differences between primary, or *de novo* GBM, and secondary GBM; the latter of which develops from lower grade II or III glioma. Primary GBM represents a majority of all malignant glioma cases (>90%) (Ohgaki and Kleihues, 2007). Primary GBM is routinely associated with mutation of PTEN (Ohgaki et al., 2004), EGFR amplification (Ekstrand et al., 1992), and p16^INK4a^ deletion (Biernat et al., 1997). Secondary GBM, which accounts for <10% of GBM diagnoses, tends to arise in younger individuals and is characterized by the presence of mutated isocitrate dehydrogenase (mIDH) and TP53 (Kohanbash et al., 2017). The median age of diagnosis for patients with a secondary GBM is ∼45 years old (Ohgaki and Kleihues, 2005). The different ages at which primary and secondary GBMs arise may reflect different microenvironmental CNS conditions that contribute to GBM cell initiation. Highlighting this plurality, GBM presenting with a mIDH is often associated with immune cell exclusion and almost totally absent of tumor cell-killing cytolytic CD8^+^ T cells (Kohanbash et al., 2017). Despite the minimal infiltration of immune cells, the presence of mIDH is associated with a favorable prognosis in GBM, as compared with primary GBM that predominantly contain wild-type IDH (Yan et al., 2009).

Our comprehensive analysis across multiple databases provides a unique perspective for assessing the risk of GBM in the elderly. While the data show interesting trends, a prospective analysis of a large patient cohort is warranted to validate the hypotheticals proposed by our current study. Some of these considerations will be built into an upcoming prospective phase I/II trial evaluating newly diagnosed GBM patients, before and after treatment with standard radiation, nivolumab (anti-PD-1 mAb) and BMS986205 (IDO1 enzyme inhibitor), led by our group. Further analysis of TCGA survival data allows for a valuable comparison of prognostic criteria between elderly and younger individuals, but still does not account for major differences of tumor biology. Future studies aimed at evaluating aging-related mechanisms and increased immunosuppression will allow for a validation of whether there are therapeutically targetable changes that potentially prevent brain tumor incidence and/or enhance the effectiveness of immunotherapeutic treatments.

Taken together, these data suggest that immunosuppressive changes in the brain are affected by processes mediated by aging and may contribute to the significantly increased brain cancer mortality rate enriched within the elderly population. The median age of a GBM diagnosis coincides with an immunosuppressive phenotype in the peripheral blood and inside the brain parenchyma. Potentially, subsets of individuals with altered expression of immunoregulatory genes possess an enhanced risk for developing GBM. A high priority must be placed on determining whether these gene expression changes contribute to tumor cell initiation and/or progression, as well as the ability to detect increases in CNS immunosuppression through a peripheral (i.e., outside of the CNS) biomarker. Further research in this area will not only allow for a better understanding of elderly GBM patient treatment, but also potentially contribute to the ability of identifying individuals at a higher risk for developing the terminal disease.

## Data Availability

All datasets generated for this study are included in the manuscript and/or the Supplementary Files.

## Author Contributions

EL performed a majority of the data mining associated with this submitted work. Data analysis was performed by EL, with statistical input from DS. All data were reviewed by DMS, MK, EAH, ETB, SO, LZ, KLL, JC, JAS, JDW, BZ, and RVL. EL and DAW prepared the figures and wrote the manuscript.

## Conflict of Interest Statement

The authors declare that the research was conducted in the absence of any commercial or financial relationships that could be construed as a potential conflict of interest.

## Footnotes

1 https://www.cdc.gov/nchs/hus/contents2017.htm#015

2 https://seer.cancer.gov/seerstat/

3 https://seer.cancer.gov/statfacts/html/common.html

4 https://costprojections.cancer.gov/graph.php#

5 http://10kimmunomes.ucsf.edu/

## Supplementary Material

FIGURE S1 GTEx Analysis. GTEx gene expression analysis of IDO1, TDO2, CD3e, and PD-L1 in the brain, pancreas, skin, and thyroid.

FIGURE S2 GTEx Analysis. GTEx gene expression analysis of CTLA-4, CD11c, PTGS2, and SLC6A4 in the brain, pancreas, skin, and thyroid.
