# Supplementary figures and images for: The Coincidence Between Increasing Age, Immunosuppression, and the Incidence of Patients With Glioblastoma
Source: Front Pharmacol. 2019 Mar 27;10:200. doi: 10.3389/fphar.2019.00200 (PMC6446059; doi:10.3389/fphar.2019.00200)

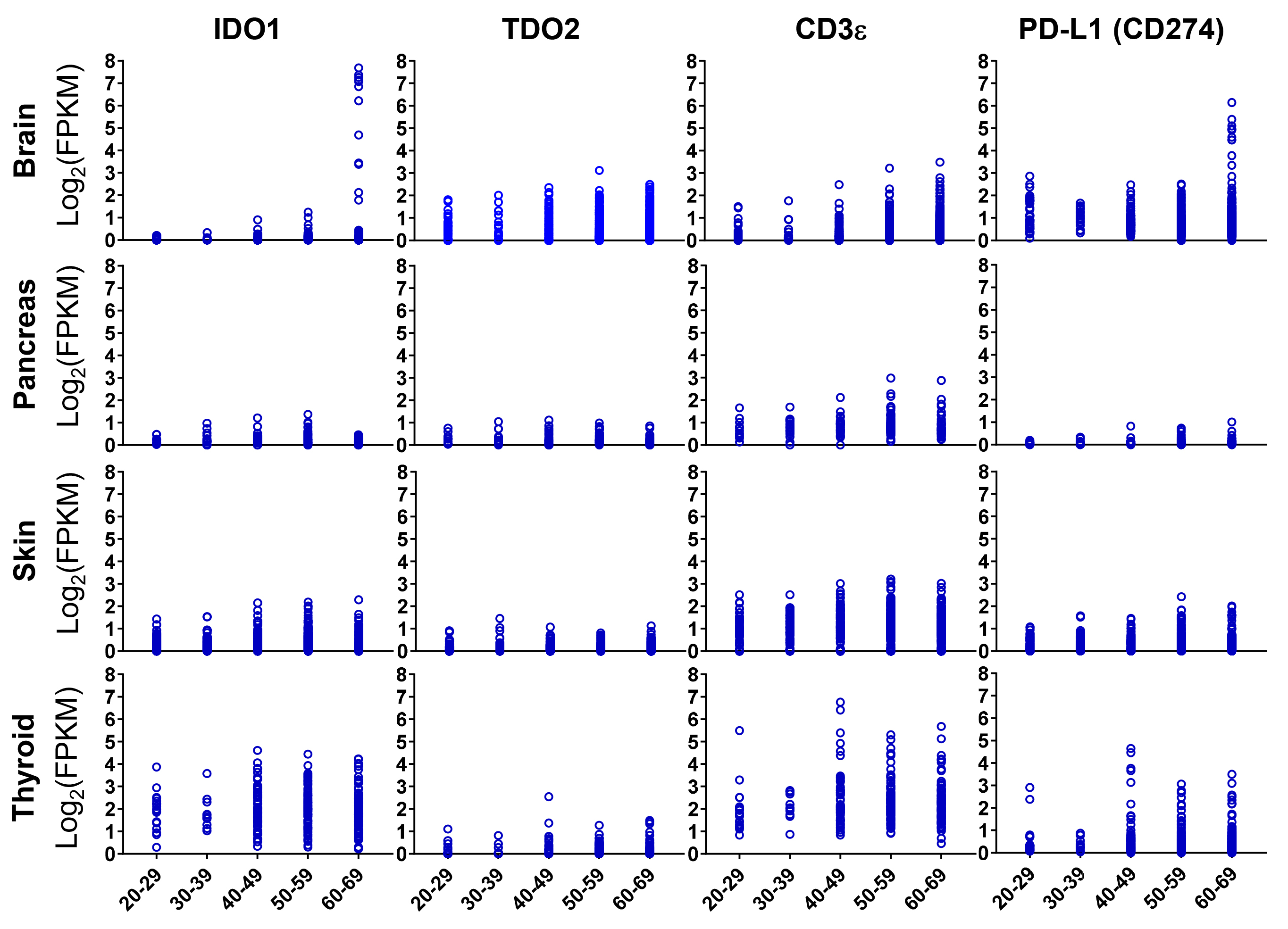

Supplement: FIGURE S1 — GTEx Analysis. GTEx gene expression analysis of IDO1, TDO2, CD3e, and PD-L1 in the brain, pancreas, skin, and thyroid. [file Image_1.jpg]

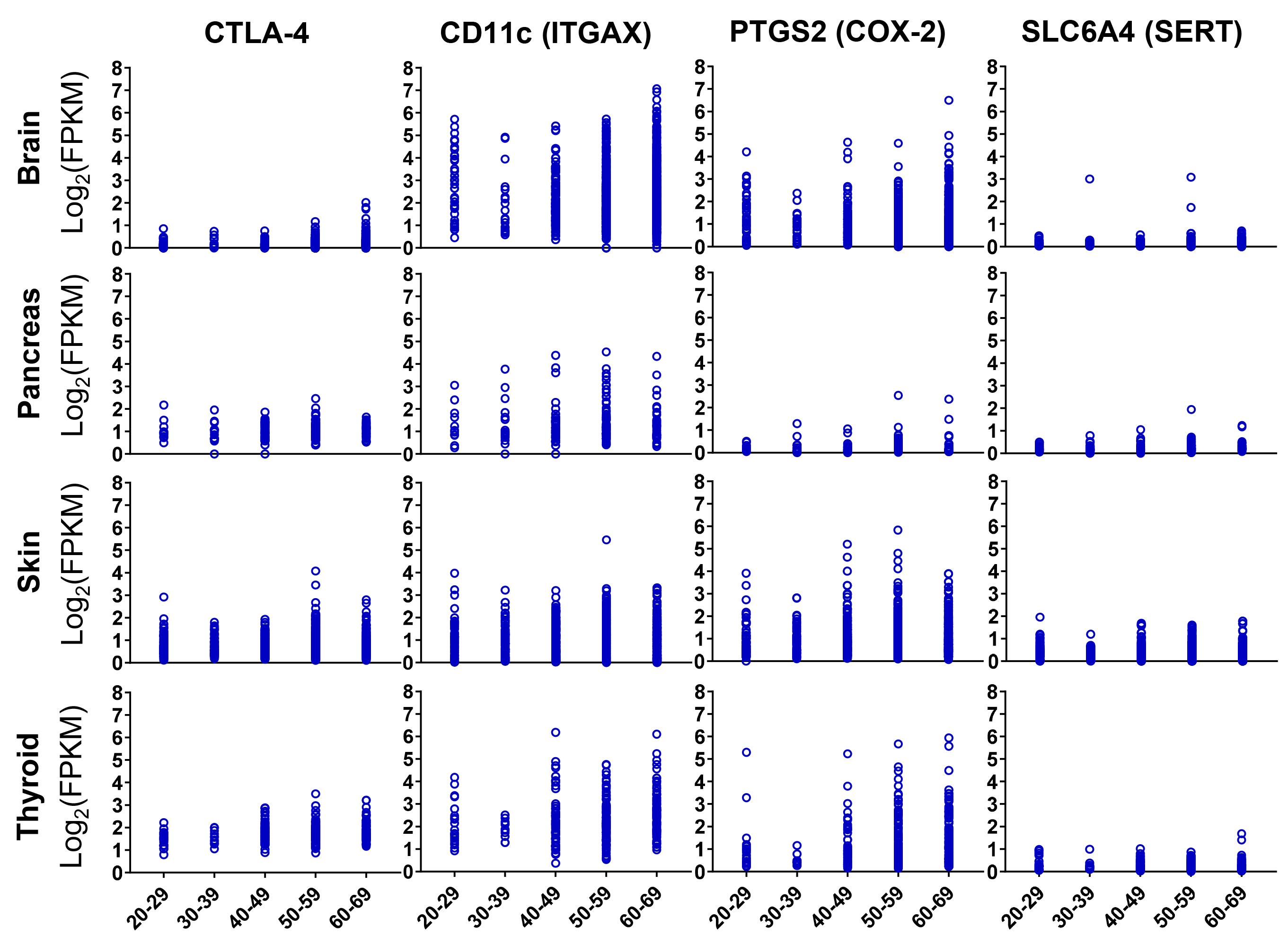

Supplement: FIGURE S2 — GTEx Analysis. GTEx gene expression analysis of CTLA-4, CD11c, PTGS2, and SLC6A4 in the brain, pancreas, skin, and thyroid. [file Image_2.jpg]
